# Baseline circulating stem-like cells predict survival in patients with metastatic breast Cancer

**DOI:** 10.1186/s12885-019-6370-1

**Published:** 2019-12-02

**Authors:** Chun-Hui Lee, Jason Chia-Hsun Hsieh, Tyler Min-Hsien Wu, Ting-Shiuan Yeh, Hung-Ming Wang, Yung-Chan Lin, Jen-Shi Chen, Chia-Lin Lee, Wen-Kuan Huang, Tsung-Min Hung, Tzu-Tsen Yen, Sheng-Chieh Chan, Wen-Chi Chou, Feng-Che Kuan, Ching-Chih Hu, Pei-Hung Chang

**Affiliations:** 1 Division of General Surgery, Department of Surgery, Chang Gung Memorial Hospital, Keelung, Taiwan; 2 Circulating Tumour Cell Lab, Division of Medical Oncology, Department of Internal Medicine, Chang Gung Memorial Hospital at Linkou, Taoyuan, Taiwan; 3 College of Medicine, Chang Gung University, Taoyuan, Taiwan; 4 Department of Chemical and Materials Engineering, Chang Gung University, Taoyuan, Taiwan; 5 Graduate Institute of Biomedical Engineering, Chang Gung University, Taoyuan City, 33302 Taiwan; 6 Department of Chemical Engineering, Ming Chi University of Technology, New Taipei City, 24301 Taiwan; 7 Division of Endocrinology and Metabolism, Department of Internal Medicine, Taichung Veterans General Hospital, Taichung, Taiwan; 8 Department of Public Health, College of Public Health, China Medical University, Taichung, Taiwan; 9 Department of Medical Research, Taichung Veterans General Hospital, Taichung, Taiwan; 10 Department of Oncology–Pathology, Karolinska Institutet, Stockholm, Sweden, Cancer Center Karolinska, Karolinska University Hospital, SE-17176 Stockholm, Sweden; 11 Department of Radiation Oncology, Chang Gung Memorial Hospital, College of Medicine, Chang Gung University, Taoyuan, Taiwan; 12 Nuclear Medicine and Molecular Imaging Center, Linkou Chang Gung Memorial Hospital, Taoyuan, Taiwan; 13 Department of Hematology and Oncology, Department of Medicine, Chang-Gung Memorial Hospital, Chiayi, 61363 Taiwan; 14 Department of Hepatogastroenterology, Chang Gung Memorial Hospital, Keelung, Taiwan; 15 Division of Hematology-Oncology, Department of Internal Medicine, Chang Gung Memorial Hospital, Keelung, Taiwan

**Keywords:** Breast cancer, Chemotherapy, Circulating tumor cells, Cancer stem cells, CD133

## Abstract

**Background:**

Circulating tumor cells (CTCs) are associated with breast cancer prognosis. Research is limited regarding the role of circulating cancer stem-like cells (cCSCs) considering the treatment response and survival among patients with metastatic breast cancer. Accordingly, we performed this prospective study to clarify the prognostic significance of baseline cCSCs for metastatic breast cancer in terms of first-line chemotherapy.

**Methods:**

Between April 2014 and January 2016, we prospectively enrolled 48 patients with stage IV breast invasive ductal carcinoma who underwent first-line chemotherapy. We identified and analyzed CTCs and cCSCs by using a protocol based on negative selection and flow cytometry before chemotherapy. CTCs were identified as EpCAM^+^Hoechst^+^CD45^–^ cells and cCSCs as CD133^+^EpCAM^+^Hoechst^+^CD45^–^ cells. cCSCs were expressed as a percentage of CTCs. The associations between CTCs, cCSCs, and the clinicopathological variables that were predictive of the treatment response and survival outcome were analyzed using univariate and multivariate analyses.

**Results:**

We identified CTCs in all the enrolled patients, with a median number of 33.9/mL CTCs. CSCs were isolated in 97.9% of the patients; the median percentage of cCSCs was 14.7%. A high baseline level of cCSCs was correlated with an inferior tumor response rate (54.2% vs. 95.8%, *p* < 0.001), overall survival (OS; median: 27.7 months vs. not reached, p < 0.001), and progression-free survival (PFS; median: 5.7 vs. 18.0 months, p < 0.001). Multivariate analysis revealed that along with other clinical variables, baseline cCSCs remained an independent prognostic factor for OS and PFS.

**Conclusions:**

Baseline cCSCs predict the treatment response as well as survival in patients with metastatic breast cancer undergoing first-line chemotherapy. Therefore, the measurement of cCSCs may assist in identifying early cancer treatment response and prognosis.

## Background

Breast cancer is a constituent of the most prevalent disease women may face [1]. Overall mortality for this disease decreased by approximately 40% from 1989 to 2015 [1]. This decrease is possibly because of the result of increased awareness, early diagnosis through screening, and major treatment advances [1]. The long-term outcomes of metastatic breast cancer may be influenced by various biological features such as the age at initial diagnosis; status of hormone receptors (HRs), including estrogen receptor (ER), and/or progesterone receptor (PR), as well as human epidermal growth factor receptor 2 (HER-2); and the numbers and sites of metastases [2, 3]. Chemotherapy, endocrine therapy, and targeted therapy remains the present standard of care is HER2-directed treatment combined with chemotherapy [4]. However, approximately 20–50% of patients do not exhibit a response to first-line treatment [5]. Moreover, the standard test for metastatic breast cancer, which involves functional and morphological imaging, does not provide sufficient prognostic information [6]. Therefore, the development of prognostic biomarkers is urgently required for patients undergoing chemotherapy to treat metastatic breast cancer.

Recently, liquid biopsy has shown promise for elucidating intratumoral and intertumoral heterogeneity. One of the most applicable liquid biopsies was established in 1869, and it is used for identifying circulating tumor cells (CTCs) as well as circulating epithelial cells (CECs) [7]. CECs and CTCs are cells that are shed into the bloodstream from the primary tumor and express epithelial cell surface (CD) markers as well as tumor-specific markers, in addition to frequently expressing epithelial markers that include the epithelial cell adhesion molecule (EpCAM) or cytokeratins [8]. Nevertheless, CECs and CTCs do not typically express red and white blood cell markers, such as CD45 and CD235 [9, 10]. These cells are believed to have the ability to metastasize to remote organs, advance thrombosis, and gain resistance to anticancer drugs [11–13]. Studies have proven the prognostic significance of CTCs in patients with assorted types of solid tumors, including those with breast cancer [14–17]. However, a novel concept of cancer stem cells has emerged, resulting in new diagnostic procedures being developed [18]. CTCs that are collected from patients with metastatic breast cancer often exhibit the overexpression of stem cell markers, suggesting that metastasis is induced by a subpopulation of CTCs that express a cancer stem cell marker [19]. Numerous researchers have revealed that a particular subdivision of CTCs can express stem cell markers (e.g., CD133 [12] or CD44 [20]), can have cells with cancer stem cell characteristics [21, 22], and can thus be considered circulating cancer stem-like cells (cCSCs) [23]. Many studies have used different definitions of cCSCs, which we have defined as cells expressing both CD133 and EpCAM after CD45 depletion. CD133 is an indicator of cells that initiate tumors in numerous cancers [24, 25]; moreover, tumor cells that express CD133 demonstrate cancer stem cell properties, characterized by self-renewal capabilities in culture, in order to differentiate into cells recapitulating initial breast cancer tumors and develop tumors in animal models [26]. Because the most commonly used marker for identifying cCSCs is CD133 [27], we included this marker to improve the ability to compare and validate our findings. cCSCs have been used to predict chemotherapy resistance in many cancers [28–30]. In a report, cCSCs were correlated with chemotherapy response and recurrence in nonmetastatic breast cancer [12]. Nevertheless, studies have yet to resolve the function of cCSCs in metastatic breast cancer, especially regarding whether these cells are associated with chemoresistance and survival. Therefore, we performed this prospective multicenter study, with the fundamental objective of evaluating the prognostic value of CTCs and CSCs in conjugation with clinical variables in patients diagnosed with metastatic breast cancer and undergoing first-line chemotherapy.

## Methods

### Study design

This prospective observational study examined the clinical importance of baseline cCSCs during first-line palliative chemotherapy for metastatic breast cancer. Our study endpoints were identifying the correlations between CTCs, CSCs, and clinicopathological variables with the treatment response rate, progression-free survival (PFS), and overall survival (OS). After determine the response to treatment, in survival analysis, we identified disease progression as well as death from any cause. In addition, we designed the analysis to be performed after more than half of the disease progression events had taken place. We report the derived study results in accordance with the guidelines outlined in the Reporting Recommendations for Tumor Marker Prognostic Studies (REMARK).

### Patient enrollment and Cancer status evaluation

This study was conducted at two medical centers: the Linkou and Keelung branches of Chang Gung Memorial Hospital, Taiwan. Our applied study protocol was approved by the Institutional Review Board of Chang Gung Memorial Hospital (approval ID: 103-0425B and 103-5322B). For protocols that required ethical approval, written informed consent was obtained from all patients. Eligible patients had breast invasive ductal carcinoma, confirmed by histologic or cytopathologic findings, that was surgically unresectable and/or metastatic (stage IV, according to the 7th edition of the staging manual of the American Joint Committee on Cancer [AJCC]). Other enrollment were included the following: (1) patients aged ≥20 years; (2) those with the ability to understand the contents of the consent form and sign it of their own accord; (3) those having satisfactory renal and liver function and blood cell counts to undergo chemotherapy; and (4) thoses who had experienced endocrine therapy failure in those with a positive HR status. We excluded patients with synchronous cancer and thoses who had cancer in the 5 years prior to enrollment. All patients were subjected to baseline evaluations, including evaluations of the clinical history, demographic data, computed tomography (CT) scan, pathological characteristics, and biochemical evaluation. For executing disease staging and management procedures used in the study, we adhered to standard treatment protocols in accordance with institutional guidelines. Systemic anticancer therapy consisted of trastuzumab plus docetaxel, paclitaxel plus gemcitabine, and doxorubicin plus cyclophosphamide, contingent upon the decision made by the physician. We executed evaluations of tumor response by using CT and/or positron emission tomography on the basis of the Response Evaluation Criteria in Solid Tumors (RECIST) version 1.1 guidelines [31]; the multidisciplinary breast tumor board ultimately decided the tumor response. PFS and OS were calculated from the date on which the first cycle of palliative chemotherapy was administered to that of disease progression or death following chemotherapy. We followed the entirety of patients until death or September 30, 2017.

### Identifying CTCs and cCSCs

**Fig. 1:**
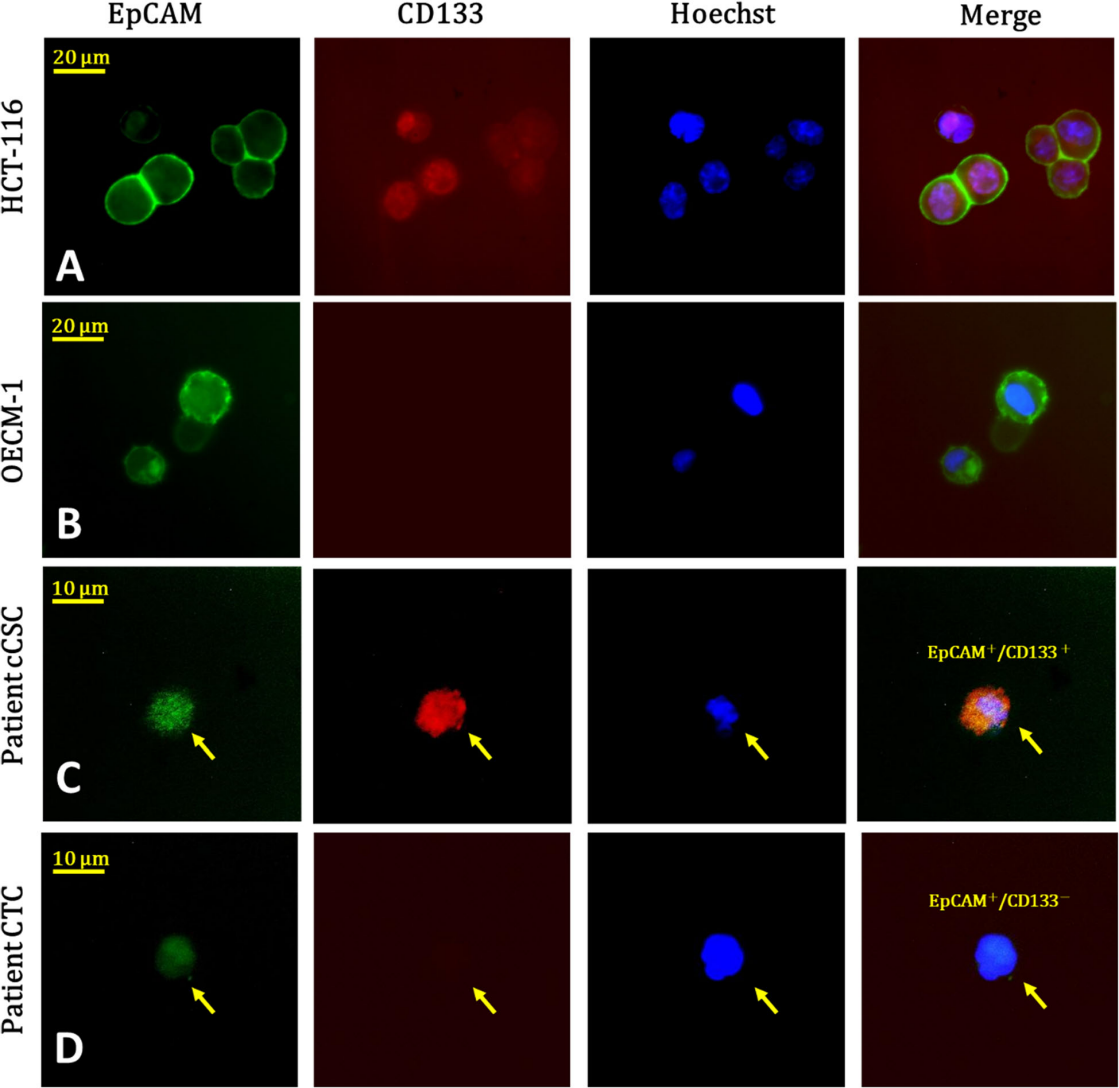
Panel (**a**) shows several cancer cells (HCT-116, an adenocarcinoma cell line) expressing CD133(red), EpCAM (green), and Hoechst (blue) as a positive control, under a fluorescence microscope. Panel (**b**) displays cancer cells (OECM-1, a squamous cell carcinoma line) expressing EpCAM (green) and Hoechst (blue), but not CD133(red), as a negative control for cCSCs. Panel (**c**) illustrates a typical cCSC expressing CD133(red), EpCAM (green), and Hoechst (blue) in the sample of one patient with cancer. Panel (**d**) reveals a typical CTC expressing EpCAM (green) and Hoechst (blue). In this study, we defined cCSCs as a subpopulation of CTCs, according to the expression of CD133.

We identified CTCs by using positive detection and negative selection strategies, and we have validated them in our previously executed studies [32, 33]. The methods adopted included the following: (1) a negative selection protocol that entails the use of a CD45 depletion kit to deplete red blood cells using lysis and leukocytes; and (2) a process of flow cytometry for quantitatively identifying CTCs (EpCAM^+^Hoechst^+^CD45^−^) and cCSCs (CD133^+^ EpCAM^+^Hoechst^+^CD45^–^), and then calculating their numbers. cCSCs were expressed as a percentage of CTCs. CTC tests were performed using peripheral blood (4 mL) after disposing of the initial 4 mL of blood, which was carried out for preventing epithelial contamination. We lysed red blood cells within 72 h, and we executed further negative selection using the EasySep Human CD45 Depletion Cocktail (25 μL/mL; STEMCELL Technologies Inc., Vancouver, BC, Canada) and EasySep Magnetic Nanoparticles (50 μL/mL; STEMCELL). Subsequently, the immunomagnetically enriched samples were subsequently spiked with OECM1/HCT116 cells, labelled using an Alexa Fluor® 488-conjugated monoclonal antibody to EpCAM (1:400; Cell Signaling Technology Inc., Danvers, MA, USA), an Alexa Fluor®647-conjugated monoclonal antibody to CD133 (1:200; CD133 [Novus Biologicals, Littleton, CO, USA]), and stained using a Hoechst 33342, blue fluorescent stain specific for DNA(20 mM; Thermo Fisher Scientific, USA). An isotype control antibody was used as an internal control, as well as the peripheral blood samples of healthy individuals (4 mL) that were and were not spiked with 1000 OECM1/HCT-166 cells, which were purchased from Taiwan’s Food Industry Research and Development Institute. Performance recovery was defined as the proportion of OECM1/HCT-116 cells detected using flow cytometry (BD FACSCalibur; BD Biosciences, San Jose, CA, USA) to the number of spiked OECM1/HCT-116 cells, and a stable coefficient of variation (CV) value has been calculated in previous studies [32, 33]. In brief, the platform can have a recovery rate of 44.6 ± 9.1% and a % coefficient of variation (CV) of 20.4%. The previous platform reported in 2015 detected 13.1 ± 0.9 cells/mL in healthy individuals (*n* = 20) [32], which was confusing and might be a background signal (i.e., a false positive result). In this revised platform, an isotype control was used for each sample, which resulted in a range of 0.0–3.0 cells/mL in healthy individuals in this study cohort (n = 20) [32, 33]. We defined CTCs as cells that tested positive for both EpCAM and Hoechst 33342. cCSCs were defined as cells expressing CD133, EpCAM and Hoechst simultaneously (Fig. 1).

### Statistical analysis

For categorical and continuous variables, we report patients’ demographic data as a number (%) and median (range), respectively. We executed univariate and multivariate analyses; in the multivariate analysis, we examined all factors used in the executed univariate analysis, but we display only those factors with statistical significance. In addition, we analyzed the associations of variables with PFS and OS by employing a multivariate Cox proportional hazards model using a forward stepwise approach (logistic regression). We executed survival analysis by using the Kaplan–Meier method; the log-rank test was used to examine any differences. Optimal categorical (cut point) data analysis was performed using the X-tile statistical package (Yale University, New Haven, CT, USA) [34]. Correlations between CTCs or cCSCs and treatment response were calculated using the Pearson chi-squared or the Fisher exact test for an expected number of < 5 per cell. We carried out the described statistical analyses with SPSS for Windows (version 18; SPSS Inc., Chicago, IL, USA). All executed statistical analyses in this study were two-sided. Moreover, we considered the observed differences to be significant when a *p* value of < 0.05 was obtained.

## Results

Between April 2014 and January 2016, we prospectively enrolled 48 patients with metastatic breast cancer before they started receiving first-line palliative chemotherapy. Table 1 presents the baseline patient characteristics. The enrolled patients’ median age was 52 years (range: 28–81 years); all the patients were women (100.0%). The performance status, determined on the basis of the Eastern Cooperative Oncology Group (ECOG) guideline was between 0 and 1 for most enrolled patients (85.4%). According to the 7th Edition AJCC criteria, all patients had stage IV disease. Thirty-five patients (72.9%) were ER and PR, and 23 (47.9%) were positive for HER-2. Eight patients (16.7%) had triple-negative breast cancer. Thirty-two patients (66.7%) had more than two metastatic sites. The bones were the most common metastatic site (72.9%), followed by the liver (37.5%), distant lymph nodes (35.4%), lung (33.3%), brain (14.6%), and pleura (12.5%). Thirty-four patients (70.8%) had visceral metastases. A depiction of cCSCs and CTCs is presented in Fig. 1. We identified CTCs in all the enrolled patients, with the median number of identified CTCs being 33.9/mL (range: 4.5–555/mL). We isolated cCSCs in 97.9% of patients, and the median proportion of CSCs was 14.7%. After a median duration of follow-up of 24.7 months (range, 0.6–43.5 months), we observed that of the 48 patients enrolled in the study, 43 (89.5%) had disease progression and 16 (33.3%) died. The median duration of PFS was 8.5 months (95% confidence interval: 5.3–11.7 months), and the median OS had not been reached by the end of the study.

**Table 1:** Basic characteristics of enrolled patients (*N* = 48)

	N	%
Age, median, years (range)	52 (28–81)	
Sex		
Female	48	100.0%
Staging (AJCC 7th Edition)		
Stage IV	48	100.0%
Performance status (ECOG)		
0–1	41	85.4%
≥ 2	7	14.6%
Receptor status		
ER and/or PR positive	35	72.9%
HER-2/neu positive	23	47.9%
Triple-negative (ER/PR/HER2)	8	16.7%
Number of metastases		
Single metastasis	16	33.3%
≥ 2 metastases	32	66.7%
Site of distant metastasis at study enrollment		
Bone	35	72.9%
Liver	18	37.5%
Distant lymph nodes	17	35.4%
Lung	16	33.3%
Brain	7	14.6%
Pleura	6	12.5%
Visceral metastasis†	34	70.8%
Nonvisceral metastasis	14	29.2%

Abbreviations: AJCC, American Joint Committee on Cancer; ECOG, Eastern Cooperative Oncology Group. SD: standard deviation; CI: confidence interval. ER, estrogen receptor; PR, progesterone receptor; HER2, human epidermal growth factor receptor 2; PFS: progression-free survival; OS: overall survival

†Visceral sites include the lungs, liver, brain, adrenal glands, and pleura (with or without effusion). Nonvisceral sites were defined as the breast, lymph nodes, chest wall, bones, and skin

**Fig. 2:**
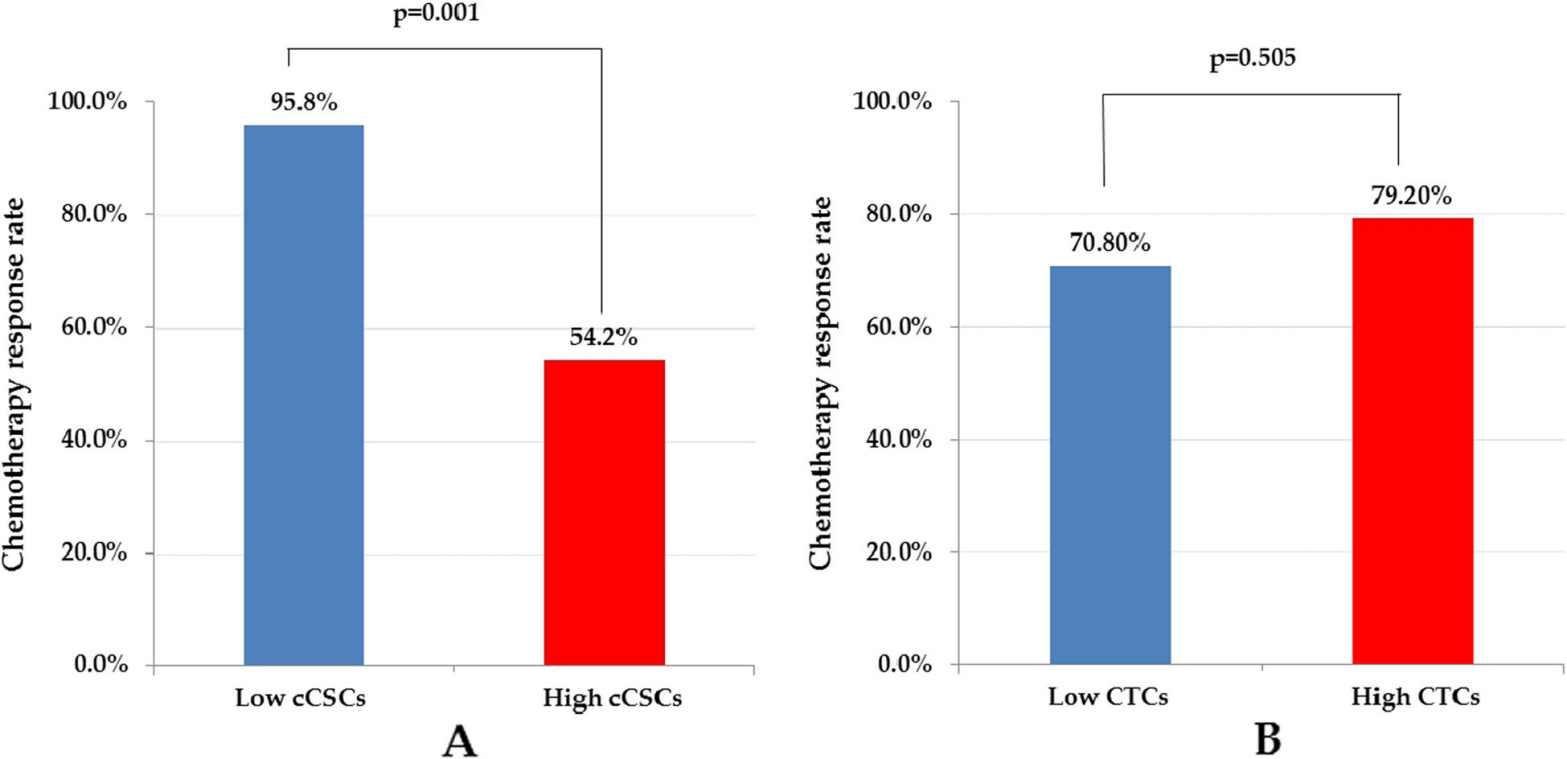
Chemotherapy response rate stratified by baseline (A) circulating tumor cells (CTCs) and (B) circulating cancer stem-like cells (cCSCs)

**Fig. 3:**
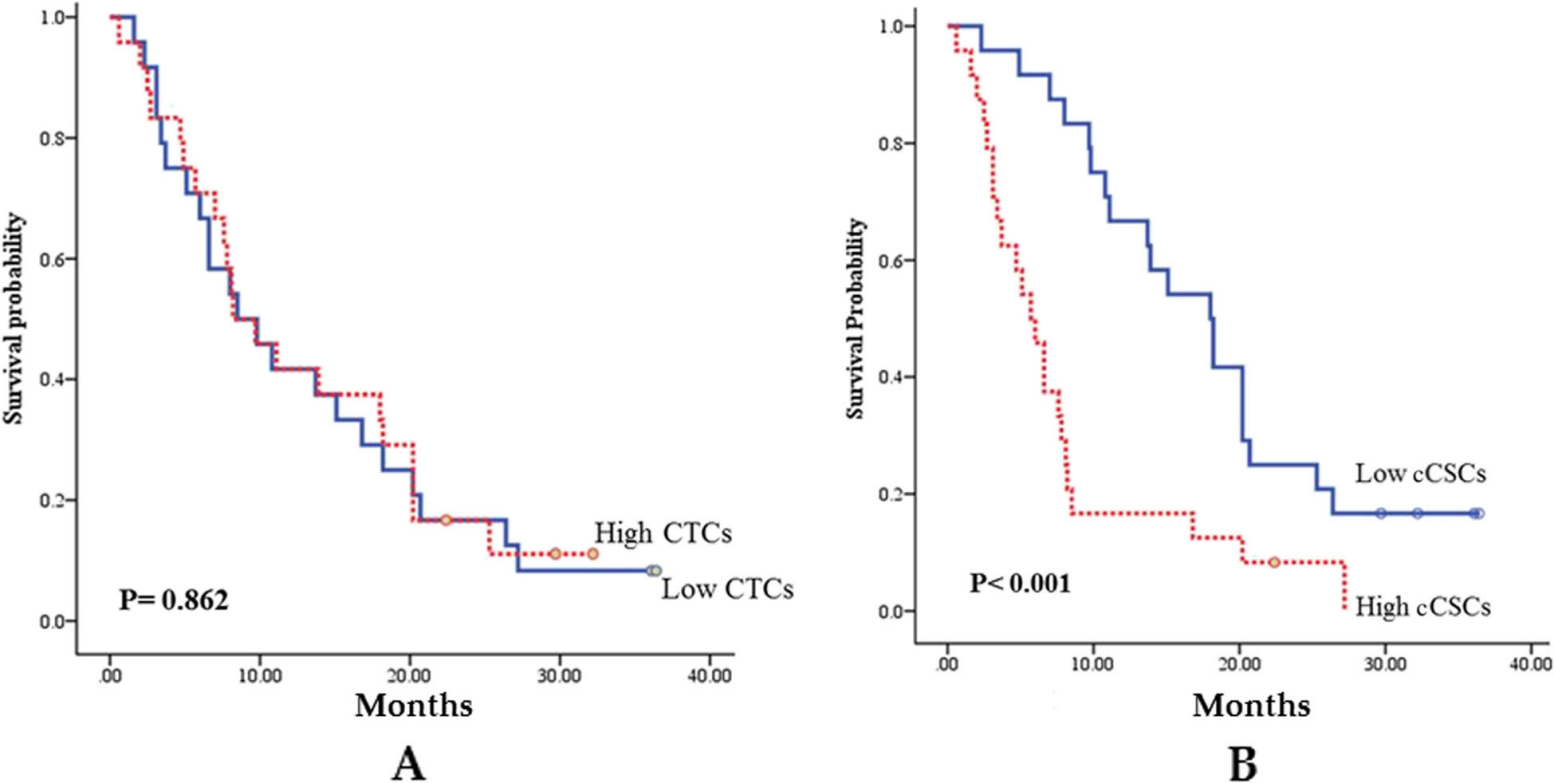
(A) Comparison of progression-free survival (PFS; months) between patients with a high circulating tumor cells (CTCs) counts (dotted line) and low CTCs counts (solid line). (B) Comparison of PFS (months) between patients with high circulating cancer stem-like cells (cCSCs) counts (dotted line) and low cCSCs counts (solid line). Censored observations are indicated as points on the curves

**Fig. 4:**
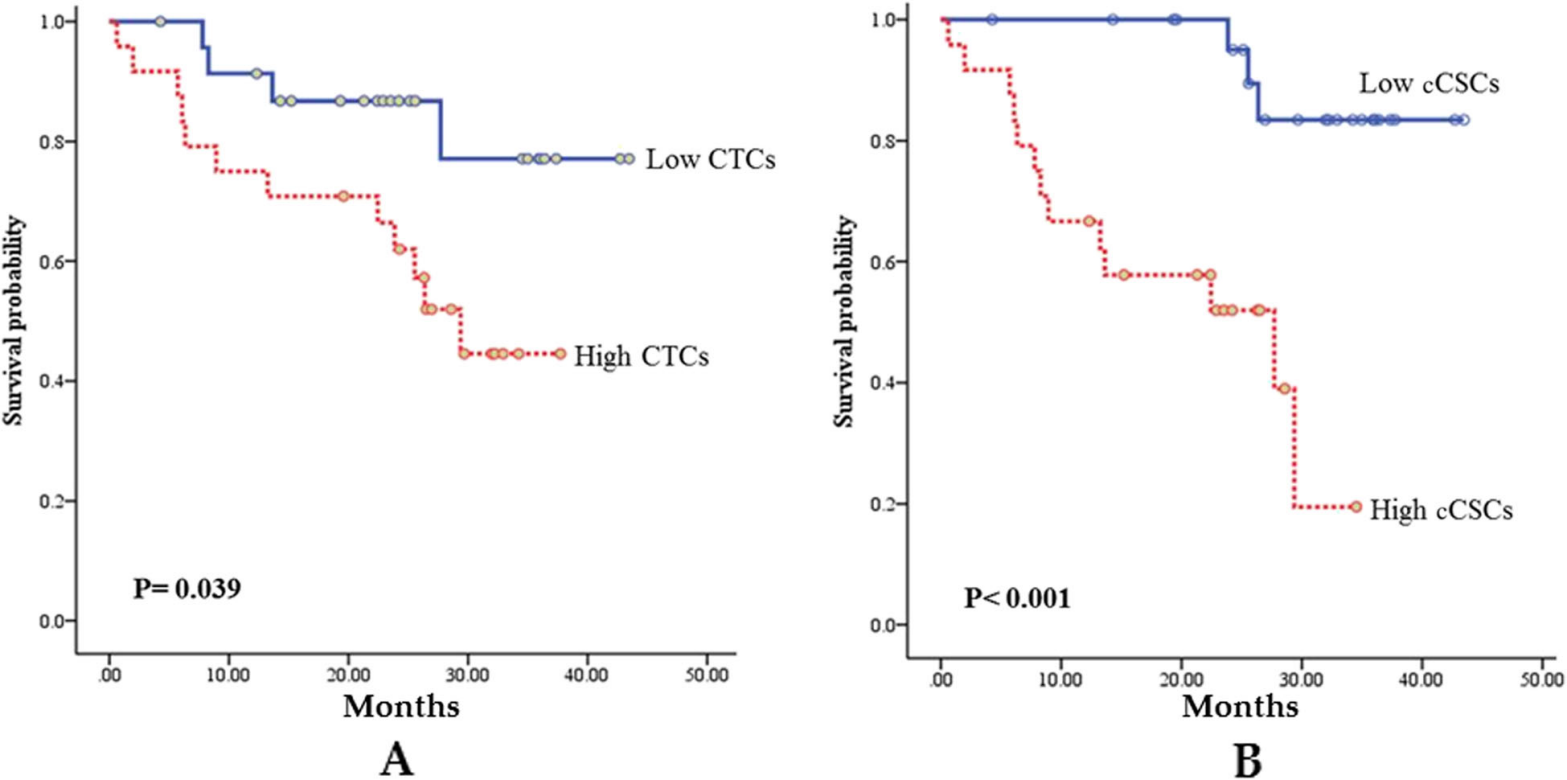
(A) Comparison of overall survival (OS; months) between patients with high circulating tumor cell (CTCs) counts (dotted line) and low CTCs counts (solid line). (B) Comparison of OS (months) between patients with high circulating cancer stem-like cell (cCSCs) counts (dotted line) and low cCSCs counts (solid line). Censored observations are indicated as points on the curves

According to a receiver operating characteristic analysis, the optimal cutoffs for high and low measurement values of CTCs and CSCs were 13.3/mL and 32.5%, respectively. No statistically significant difference was observed between the group with low CTCs and that with high CTCs regarding the chemotherapy response rate (79.2% vs. 70.8%, *p* = 0.505) after 3 months of treatment (Fig. 2A) and progression-free survival (PFS; 8.5 months vs. 8.2 months, *p* = 0.862; Fig. 3A). Patients with low CTCs counts had a better overall survival (OS) than did those with high CTCs counts (18.9 months vs. 5.7 months, *p* < 0.001; Fig. 4A). However, a low cCSCs count was correlated with a superior tumor response rate (95.8% vs. 54.2%, p < 0.001; Fig. 2B), PFS (median, 18.0 vs. 5.7 months, p < 0.001; Fig. 3B), and OS (median, not reached vs. 27.7 months, p < 0.001; Fig. 4B), compared with a high cCSCs count. In combination with other clinical parameters, a multivariate analysis showed that ECOG performance status, triple-negative (ER/PR/HER2) type, and baseline cCSCs were independent prognostic factors for PFS. Baseline CTCs and cCSCs were independent prognostic factors for OS (Table 2).

**Table 2:** Univariate and multivariate analyses for progression-free and overall survival

	PFS				OS			
Parameters	Univariate		Multivariate		Univariate		Multivariate	
	HR (95% CI)	p	HR (95% CI)	p	HR (95% CI)	p	HR (95% CI)	p
Age > 65 y	1.007 (0.983–1.032)	0.584			1.001 (0.960–1.004)	0.968		
ECOG PS > 1	4.441 (1.948–10.127)	< 0.001	3.544 (1.413–8.889)	0.005	3.390 (1.161–9.898)	0.026		
Visceral metastasis†	1.410 (0.723–2.752)	0.314			3.473 (0.785–15.386)	0.101		
Triple-negative (ER/PR/HER2)	4.463 (1.900–10.483)	0.001	3.329 (1.328–8.342)	0.010	3.995 (1.177–13.296)	0.026		
CTCs > 32/ml	1.003(1.000–1.006)	0.089			1.006 (1.003–1.010)	< 0.001	7.266 (1.775–29.741)	0.006
cCSC > 15%	2.903 (1.563–5.392)	0.001	2.867 (1.338–6.140)	< 0.0001	8.236 (2.269–29.894)	0.001	16.238 (3.167–83.264)	0.001

**Abbreviations**: OS: overall survival; PFS: progression-free survival; HR: hazard ratio; ECOG PS: Eastern Cooperative Oncology Group performance wtatus; ER, estrogen receptor; PR, progesterone receptor; HER2, human epidermal growth factor receptor 2; CTCs: circulating tumor cells; cCSCs: circulating cancer stem cells

## Discussion

On the basis of evidence indicating that CD133 is a stem cell marker [12], we hypothesized that cCSCs, which are CD133-expressing CTCs, have a role in chemotherapy resistance and breast cancer prognosis. We therefore performed the current prospective multicenter study that evaluated 48 patients with unresectable and/or metastatic stage IV breast cancer undergoing first-line palliative chemotherapy. We revealed several elementary but crucial findings in terms of the function of CTCs as well as cCSCs in metastatic breast cancer. We demonstrated that patients with high baseline cCSCs before chemotherapy had inferior outcomes in terms of chemotherapy response, PFS, and OS. High baseline CTCs also predicted less favorable OS, but this was not associated with chemotherapy response or PFS. In the multivariate analysis, poor performance status, triple-negative type, and baseline cCSCs were independent prognostic factors for PFS. However, baseline CTCs and CSCs were independent predictors of OS.

The CTC quantity has emerged as a potential biomarker, and the presence of CTCs in peripheral blood was reported to be correlated with a relatively poor prognosis of metastatic breast cancer [35]. In the current study, pretreatment CTCs remained an independent prognostic factor for OS, a finding that was in line with those of related studies [35–37]. However, in the current study, performance status, triple-negative type, and baseline cCSCs, rather than CTCs, were independent prognostic factors for PFS, which partly contradicts the findings of relevant studies [35–38]. Some plausible reasons why the CTC number influences OS rather than the first PFS include (i) the limited sample size (ii) the fact that chemotherapy regimens were not the same in the entire cohort; and (iii) breast cancer patients in real-world settings would possibly receive multiple combination treatments, such as local palliative radiotherapy, which might alter the first PFS but not OS in this cohort. The most useful prognostic factors in clinical settings for breast cancer are the performance status, age, site of metastasis (presence of visceral metastasis or not), and HR and HER2 status [39, 40]. Patients having a poor performance status may not tolerate intensive chemotherapy, and such patients may experience high toxicity during chemotherapy [41]. Of the patients included in the current study, 14.6% had a relatively poor performance status (≥2). Clinically, it is not surprising that a poor performance status is associated with poor PFS, which was also demonstrated in our study. Nevertheless, the prognosis of patients diagnosed as having triple-negative breast cancer was reported to be poorer than that of those diagnosed as having other subtypes of breast cancer [42], which is also in accordance with our study results. However, in addition to the clinical prognostic factors, baseline cCSCs remained an independent prognostic factor for OS and PFS, suggesting a more powerful role of this subset of CTCs.

Research has analyzed the role of cCSCs in many cancers, which have been examined using RT-PCR [27, 43], enumeration with a gradient protocol [44, 45], and flow cytometry [46, 47]. The results of such research have indicated that cCSCs are linked to an unfavorable prognosis in various cancers, including colorectal cancer [27, 48], gastric cancer [45], lung cancer [46], prostate cancer [49], head and neck cancer [50] and hepatocellular carcinoma [43]. Nadal et al. [12] revealed the detection of a relative enrichment of CD133-expressing cells in nonluminal tumor subtype nonmetastatic breast cancer after chemotherapy, indicating a possible role of CD133-expressing CTCs in processes of chemotherapy resistance; however, those authors did not analyze the prognostic role of such CTCs. According to our review of the relevant literature, our research is the first prospective study to examine the prognostic significance using a single blood sample that was obtained from patients having metastatic breast cancer undergoing first-line chemotherapy, showing that baseline cCSCs is an independent prognostic factor for survival, in combination with the other clinical prognostic factors.

A reliable method developed for detecting or isolating CTCs could act as a valuable predictor before anticancer treatment is administered in patients with cancer. Although a previously developed CellSearch® system was granted approval by the US Food and Drug Administration (FDA) in 2004, it is unavailable in most hospitals. To date, no standard protocol or method is available for identifying or isolating CTCs. This is largely owing to the relatively low detection efficiency and high cost of each test. In 2015, our team developed a negative selection strategy combined with flow cytometry for the identification of CTCs with multiple surface marker expressions, which has received validation for numerous cancer types [14, 16, 32]. Compared with the only FDA-approved device, CellSearch®, the method used in this study has demonstrated a similar recovery rate and better detection rate in late-stage cancer settings [35]. Crucially, CTC testing based on the negative selection strategy has a greater likelihood of preserving cells without cytokeratins or EpCAM expression [8, 51, 52]. These cells should be preserved during isolation processes, and the negative selection protocol offers the most favorable opportunity for this. In the current study, we detected CTCs in all patients and isolated cCSCs in 97.9% of patients, indicating a high detection rate. Our platform provides a superior detection rate and might have more success in identifying more CTCs, to determine a cutoff of clinical importance. The cutoff value for the number of CTCs and cCSCs determined using receiver operating characteristic curves was also analyzed as an independent prognostic factor for survival. On the basis of this straightforward and inexpensive method, we posit that analyzing the CTC and cCSC ratios through flow cytometry combined with a negative selection strategy, as done in the current study, represents a widely available method that can be used to determine predictive and prognostic factors in patients undergoing first-line palliative chemotherapy for metastatic breast cancer. The methodology of CTC detection still requires standardization and an automatic device, to maintain a stable CV. The method used in the current study involves operator-dependent analysis, although this approach could be applied in a small laboratory, such as those in most hospitals. Consensus on the standardization of protocols is still required.

Certain limitations should be acknowledged when evaluating this study. First, the study had a small number of participants, which limits the strength of our results. However, estimating an ideal sample size in a biomarker study is difficult; also, the sensitivity of the tool used in the current study had not been reported or tested in the same population previously. The lack of sensitivity highlights the limitation of the sample size estimation. Therefore, this study should be viewed as an exploratory study, even though our results are statistically significant. Second, the chemotherapy regimen was inconsistent among patients; therefore, the analysis of the baseline CTCs and CSCs associated with survival might have been confounded by different treatment regimens. Third, although we used a clear and strict definition of cCSCs, we did not isolate these cells to confirm that they possessed tumorigenicity in vivo, invasion, or self-renewal, which is why we refer to them as stem-like cells. Third, we did not include cytokeratins (CK) as one of the markers given the fact that fixation and permeabilization processes commonly lose cells, which is the main goal we want to avoid in the negative selection strategy. Fourth, we did not perform CD45 characterization in the downstream analysis, which might cause some bias in CTC identification. Therefore, larger independent trials are required to validate our findings.

## Conclusions

We identified cCSCs at baseline palliative chemotherapy as an independent prognostic factor for predicting survival outcomes such as OS, and PFS, as well as tumor response in patients diagnosed as with metastatic breast cancer. We believe that these findings facilitate the identification of patients with the least favorable prognoses and treatment outcomes in clinical settings.

## Supplementary information

**Additional File 1.** Correlations among baseline patient characteristics and baseline cCSC ratio. Abbreviations: cCSCs: circulating cancer stem cells; AJCC, American Joint Committee on Cancer; ECOG, Eastern Cooperative Oncology Group; SD: standard deviation; CI: confidence interval; HR, hormone receptor; HER2, human epidermal growth factor receptor 2; PFS: progression-free survival; OS: overall survival. †Visceral sites include the lungs, liver, brain, adrenal glands, and pleura (with or without effusion). Nonvisceral sites were defined as the breast, lymph nodes, chest wall, bones, and skin.

## Data Availability

The datasets used or analyzed during the current study are available from the corresponding author on reasonable request.

## Abbreviation

cCSCs: Circulating cancer stem-like cells
CD: Cell surface
CECs: Circulating epithelial cells
CT: Computed tomography
CTCs: Circulating tumor cells
ECOG: Eastern Cooperative Oncology Group
EpCAM: Epithelial cell adhesion molecule
ERs: Estrogen receptors
HER-2: Human epidermal growth factor receptor 2
HR: Hormone receptors
OS: Overall survival
PFS: Progression-free survival
PR: Progesterone receptors
