# Supplementary material for: Baseline circulating stem-like cells predict survival in patients with metastatic breast Cancer
Source: BMC Cancer. 2019 Dec 2;19:1167. doi: 10.1186/s12885-019-6370-1 (PMC6889331; doi:10.1186/s12885-019-6370-1)
Supplement: Supplementary file 1 — Additional File 1. Correlations among baseline patient characteristics and baseline cCSC ratio. Abbreviations: cCSCs: circulating cancer stem cells; AJCC, American Joint Committee on Cancer; ECOG, Eastern Cooperative Oncology Group; SD: standard deviation; CI: confidence interval; HR, hormone receptor; HER2, human epidermal growth factor receptor 2; PFS: progression-free survival; OS: overall survival. †Visceral sites include the lungs, liver, brain, adrenal glands, and pleura (with or without effusion). Nonvisceral sites were defined as the breast, lymph nodes, chest wall, bones, and skin. [file 12885_2019_6370_MOESM1_ESM.docx]

|  | **N** | **%** | **Baseline cCSC ratio (mean±SD) (%)** | **P value** |
| --- | --- | --- | --- | --- |
| **Age**, years, median (range) | 52 (28–81) | |  |  |
| **Sex** |  |  |  |  |
| Female | 48 | 100.0% | 24.4±25.9 | - |
| **Staging (AJCC 7th Edition)** |  |  |  |  |
| Stage IV | 48 | 100.0% | 24.4±25.9 | - |
| **Performance status (ECOG)** | | |  |  |
| 0–1 | 41/7 | 85.4%/14.6 | 19.7±22.8/45.0±29.7 | 0.007 |
| **Receptor status** | | |  |  |
| HR positive/negative | 35/13 | 72.9%/27.1% | 19.2±22.1/38.2±30.1 | 0.058 |
| HER2/neu positive/negative | 23/25 | 47.9%/52.1% | 22.9±25.5/25.8±26.7 | 0.706 |
| Triple-negative/Non triple-negative | 8/40 | 16.7%/83.3% | 46.4±32.4/20.0±22.4 | 0.007 |
| **Number of metastases** |  |  |  |  |
| Single metastasis/≥2 metastases | 16/32 | 33.3%/66.7% | 23.8±6.2/26.9±4.7 | 0.435 |
| **Site of distant metastasis at study enrollment** |  |  |  |  |
| Visceral metastasis†/Nonvisceral metastasis | 34/14 | 70.8%/29.2% | 28.8±27.3/13.8±18.8 | 0.037 |

Additional File 1. Correlations among baseline patient characteristics and baseline cCSC ratio

Abbreviations: cCSCs: circulating cancer stem cells; AJCC, American Joint Committee on Cancer; ECOG, Eastern Cooperative Oncology Group; SD: standard deviation; CI: confidence interval; HR, hormone receptor; HER2, human epidermal growth factor receptor 2; PFS: progression-free survival; OS: overall survival

†Visceral sites include the lungs, liver, brain, adrenal glands, and pleura (with or without effusion). Nonvisceral sites were defined as the breast, lymph nodes, chest wall, bones, and skin.
